# Genetic Determinants of Fiber-Associated Traits in Flax Identified by Omics Data Integration

**DOI:** 10.3390/ijms232314536

**Published:** 2022-11-22

**Authors:** Alexander Kanapin, Tatyana Rozhmina, Mikhail Bankin, Svetlana Surkova, Maria Duk, Ekaterina Osyagina, Maria Samsonova

**Affiliations:** 1 Centre for Computational Biology, Peter the Great St. Petersburg Polytechnic University, 195251 St. Petersburg, Russia; 2 Laboratory of Breeding Technologies, Federal Research Center for Bast Fiber Crops, 172002 Torzhok, Russia; 3 Mathematical Biology & Bioinformatics Laboratory, Peter the Great St. Petersburg Polytechnic University, 195251 St. Petersburg, Russia; 4 Theoretical Department, Ioffe Institute, 194021 St. Petersburg, Russia

**Keywords:** flax, GWAS, fiber, GEMMA, gene expression

## Abstract

In this paper, we explore potential genetic factors in control of flax phenotypes associated with fiber by mining a collection of 306 flax accessions from the Federal Research Centre of the Bast Fiber Crops, Torzhok, Russia. In total, 11 traits were assessed in the course of 3 successive years. A genome-wide association study was performed for each phenotype independently using six different single-locus models implemented in the GAPIT3 R package. Moreover, we applied a multivariate linear mixed model implemented in the GEMMA package to account for trait correlations and potential pleiotropic effects of polymorphisms. The analyses revealed a number of genomic variants associated with different fiber traits, implying the complex and polygenic control. All stable variants demonstrate a statistically significant allelic effect across all 3 years of the experiment. We tested the validity of the predicted variants using gene expression data available for the flax fiber studies. The results shed new light on the processes and pathways associated with the complex fiber traits, while the pinpointed candidate genes may be further used for marker-assisted selection.

## 1. Introduction

Flax fibers are used as a textile raw material for production of cords, weaving yarn, fashionable garments and high-quality fabric upholstery [1,2]. Since the 1930s, flax fibers have become an important reinforcement component in composite materials due to its unique mechanical properties [3,4]. Currently, composites made of flax fibers are widely used in the automotive industry [5]. The average mechanical performance of flax fibers is very close to that of glass, however, contrary to glass-based composites, flax fibers have reduced environmental impact when incorporated into composite materials. [5].

An elementary fiber is an individual sclerenchyma cell with an exceptionally thick cell wall and an extreme length to diameter ratio (above 1000) [4]. The cells originate from the apical meristem and develop in two stages: elongation and wall thickening. Initially, the elongation of flax fibers proceeds synchronously with neighboring cells; however, after several hours an intrusive growth begins, leading to fibers that reach several centimeters in length. The cell wall thickening includes the deposition of several new layers from the inside of the primary cell wall, and it begins when elongation stops. The first additional layer (so-called S layer) is very thin, while the further deposited layers are characterized by high cellulose content, axial orientation of cellulose microfibrils, almost complete absence of xylan and lignin and the presence of rhamnogalacturonan-I [6,7,8]. Flax fibers are gathered in bundles located at the periphery of the stalk, providing for its reinforcement [1,9]. Each bundle is composed of 15–50 elementary fibers embedded in a gel-like matrix and joined together.

A technical quality of the fiber is a complex trait defined by plant genotype, environment and after-harvest processing. The fiber traits manifest a significant degree of variation between cultivars [3,4,10]. The most important morphological traits associated with the fiber quality are the following: (a) technical stem length (from the bottom of the stem to the first floral ramification), (b) number of internodes and (c) stem diameter. Flax cultivars with a longer technical stem have compact, dense bast bundles constructed of long elementary fibers. Plants with long internodes and a small number of leaves have a higher fiber quality, as the fiber bundles are torn at the attachment point of the leaf blades. Cultivars with thicker stems have sparse and enlarged fiber bundles with well-developed woody cores, thus producing coarse and less flexible fiber with low spinning quality. The cylindrical shape of the stalk indicates a uniform distribution of the elementary fibers along its length, which ensures homogeneous fiber maturation and even color distribution [10].

There are two common approaches to harness phenotypic variability for dissection of the genetic architecture of agronomic traits. Linkage mapping is based on the analysis of progeny from biparental crosses, and its power predominantly relies on recombination events taking place in the F1 generation. This limits the practical application of linkage mapping for breeding, as the estimated marker effects are specific to the same or genetically related populations and are often not transferable to other genetic backgrounds [11]. GWAS, on the contrary, exploits natural variation and the long history of recombination events captured in large germplasm collections. Inbred plant species such as flax have the practical effect of immortalizing the genotype, providing an unlimited supply of identical seeds that can be grown and assayed in replications for a variety of traits and under various conditions.

Nevertheless, in spite of the increase in size of GWAS populations, most GWAS analyses still have unsignificant statistical power to detect associations with alleles of small effect and low frequency that collectively explain a large fraction of the trait heritability. One of the approaches to improve the power of GWAS is to combine the multiple related traits in a multivariate framework [12]. Multitrait GWAS increases statistical power by accounting for trait correlations and pleiotropic effects of polymorphisms, as well as by aggregating weak genetic effects across traits [13]. Additional advantages of multitrait GWAS in comparison with a standard univariate approach lie in its ability to complement missing information in one of phenotypes with the other phenotypes and to capture indirect genetic effects when an SNP affects one phenotype through its effect on the other functionally related phenotype [14,15].

Recently, numerous studies on dissecting the genetic architecture of agronomic traits in flax have been reported. Most of these analyses interrogated traits related to yield, phenology and fatty acid content [16,17,18,19,20,21,22,23,24]. Regions associated with plant height were also identified in several analyses [17,20,25,26]. Other fiber-related traits received less attention. The current information on genomic regions associated with fiber quality and content is not only limited but is also quite inaccurate, as the studies employ reduced representation sequencing methods and, in most cases, use the biparental crosses. Several studies analyzing RIL populations from crosses between linseed and fiber flax parents report QTLs associated with the following phenotypes: fiber yield and fiber content [25], fiber technical length [26], straw weight and cell wall [27]. Another study of 224 flax samples and 34,932 SFAF markers used GLM and MLM models and identified four SNP loci, of which two were associated with fiber percentage and two with fiber technical length [28].

In addition to GWAS, several high-throughput transcriptomics studies suggested candidate genes involved in fiber-intrusive elongation and tertiary cell wall deposition [8,29,30]. However, these studies were confined to a limited number of cultivars that preclude generalization due to varietal variation. Therefore, to gain an ultimate understanding of mechanisms defining fiber quality, and to prioritize genes for further in-depth experiments, it is important to make the most of the available genomic and genetic resources, as well as the analytical approaches such as GWAS.

Here, we explore the genetic control of fiber traits by mining 306 flax accessions maintained at the Federal Research Centre of the Bast Fiber Crops (Tables S12 and S13). The dataset was whole-genome sequenced at 10× depth coverage to characterize genetic diversity and population structure, as well as thoroughly phenotyped [10,31]. An in-depth characterization of genetic regions controlling fiber traits could provide a foundation for further genomic selection and breeding efforts aimed at the development of cultivars with superior fiber quality.

## 2. Results and Discussion

### 2.1. Phenotypic Variation

All traits showed abundant variation among 306 accessions (Table S1, Appendix A), substantiating GWAS application for detection of genomic regions involved in fiber control. The most variable traits are weight-related characteristics: under three different plantings (in 2019 and two times in 2020), the maximum coefficient of variation for fiber weight (FW) was 54.7% and for technical part length (TW) 43.1%. The variations in stem slenderness (SSI) and tapering (STI) indices were also among the highest, reaching 35.3% and 36.5% in 2019Y, respectively. Compared to variation in indices values, the variation coefficient for elementary fiber length (EFL) was about two times smaller, ranging between years from 12.2% to 15.8%.

Within planting comparisons, we observed statistically significant superiority of most fiber traits when fiber genotypes were compared with linseed genotypes and fiber cultivars were compared with landraces. Non-significant differences were also observed in juxtaposition of fiber cultivars and landraces for stem diameter (SD) in 2020Y-1 and 2020Y-2, distance between internodes (DI) in 2020Y-1 and stem slenderness index (SSI) in 2019Y (Table S2).

**Figure 1:**
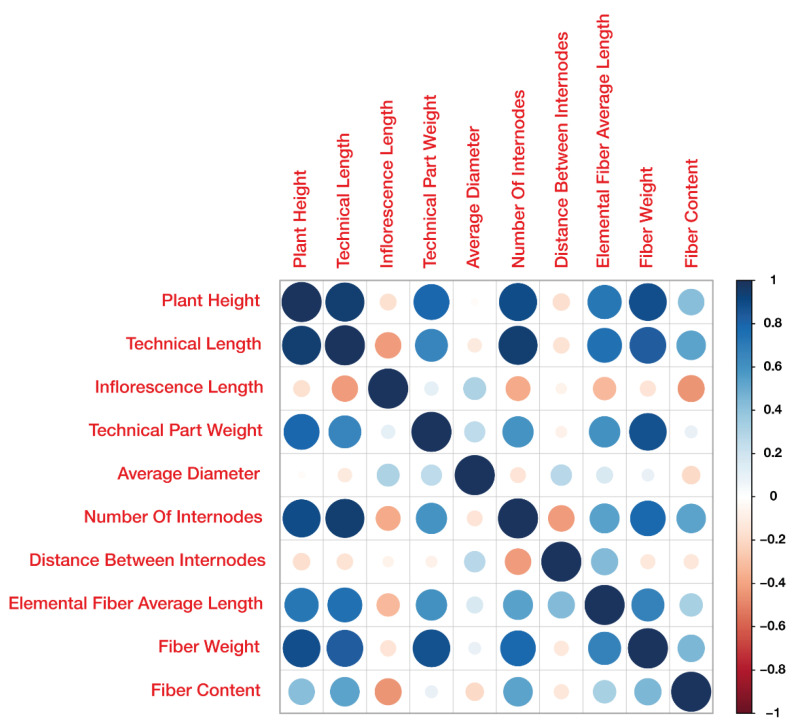
Pearson correlation between traits in 2019Y.

Most fiber-related traits are correlated (Table S3, Figure 1). A notable negative correlation was observed between stem diameter (SD) and fiber content (FC). The highest positive correlation was detected in pairwise comparisons between elementary fiber length (EFL), technical stem length (TL), plant height (PH) and the number of internodes (NI). When estimating trait correlation between different plantings, no correlation was revealed for stem tapering index (STI) and distance between internodes (DI), with small positive correlation for stem diameter (SD) and moderate positive correlation for technical part weight (TW) (Table S4). All other traits had between-planting correlation coefficient values higher than 0.7.

### 2.2. Association Mapping of Fiber Traits

After SNP calling and filtering with thresholds for an MAF of 0.05 and a call rate of 0.85, a total of 72,526 variants remained. Principal component analysis did not produce a clear separation of linseed and fiber genotypes (not shown).

**Figure 2:**
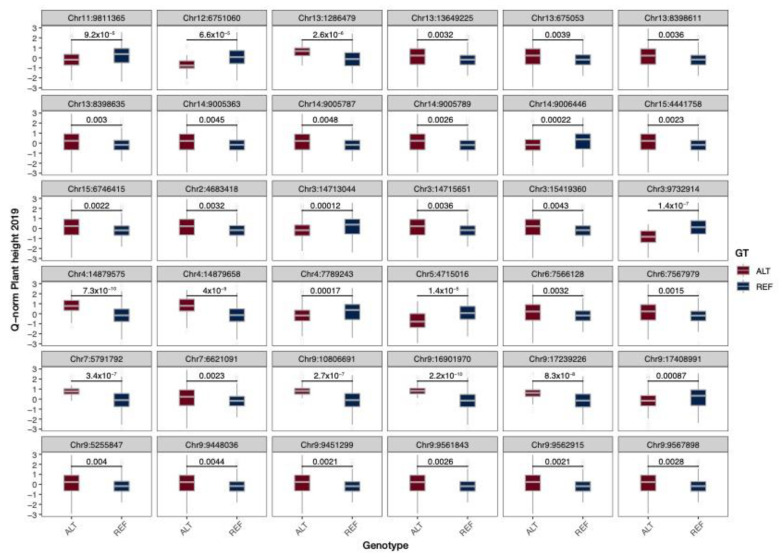
Box plots of allelic effects for representative QTNs associated with plant height in 2019. Numbers are *p*-values according to the Mann–Whitney–Wilcoxon non-parametric test.

A univariate GWAS analysis was performed using the GLM, MLM, CMLM, FarmCPU, SUPER and Blink models implemented in the GAPIT3 R package [32]. Proceeding from the GAPIT estimation of Bayesian information content, we retained five principal components to be used in GWAS. The six aforementioned methods yielded 1059, 2102 and 3170 QTNs in 2019, 2020Y-1 and 2020Y-2 plantings (FDR adjusted *p*-value 0.05, see Table S5). All models performed well in controlling population structure and family relatedness, as *p*-values deviated from the expected values at the end of distribution in all Q-Q-plots. All QTNs were discovered by at least two models. A total of 873 QTNs were detected in two years (Table S6), while 4 QTNs were found in all plantings. A total of 717 QTN detected in at least two plantings were pleiotropic. Among pleiotropic QNTs, the most often found association was with plant height, being found in 346 of 717 cases. The majority of the identified QTNs demonstrated significant allelic effect across all three plantings, as is shown in Figure 2, Figures S1 and S2 for plant height (PH). Negative alleles decrease the plant height, while positive alleles increase it.

Multitrait analysis was conducted with GEMMA [33] package for five traits, namely, plant height (PH), technical stem length (TL), number of internodes (NI), fiber content (FC) and fiber weight (FW). The phenotype values have been normalized with quantile normalization for each phenotype, and SNPs were filtered with the following thresholds: MAF 0.05 and call rate 0.85. We used linear mixed model and score test for GEMMA calculation, and the threshold for false discovery rate was set at 0.05. Finally, 162 QTNs were found, which occur in all three plantings and satisfy the FDR threshold (Table S7).

Corrected to chromosome-specific LD, several QTNs detected by GEMMA and GAPIT models coincided (Table S8a). With whole-genome sequencing information and advanced statistical methods at hand, we were able to achieve higher precision in QTL detection in comparison with previous studies based on the reduced representation sequencing methods, identification of SNPs without a reference sequence and biparental crosses. Nevertheless, several QTNs detected by us were in close proximity to QTLs predicted from biparental crosses [25,26,27] (Table S8b), providing for general agreement between the studies.

### 2.3. Fiber-Related Candidate Genes

A two-stage procedure was introduced to pinpoint fiber-related candidate genes. First, we defined intervals centered on stable QTNs detected in at least two plantings and set according to LD decay estimated for each chromosome (Table S9). We called such intervals QTN loci. In total, we found 1645 genes and 1105 genes in QTN loci detected with GAPIT3 and GEMMA, respectively.

**Figure 3:**
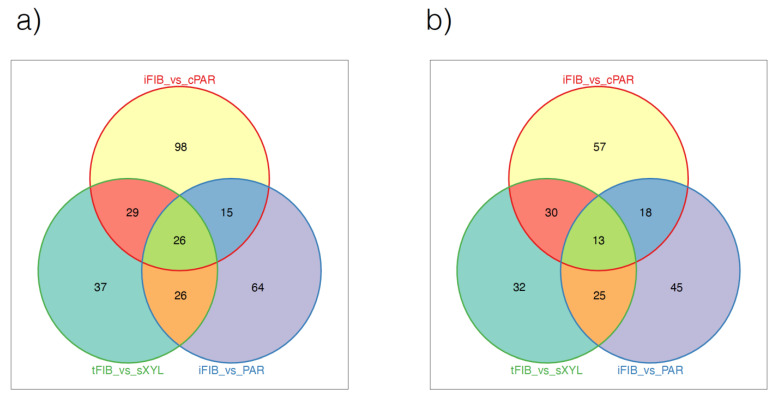
Venn diagram representing the number of differently expressed genes in QTN loci detected with GAPIT (**a**) and GEMMA (**b**).

Next, we checked the expression of these genes using transcriptome libraries from four flax tissues: bast fibers isolated at the stage of intrusive elongation (iFIB) or at the stage of tertiary cell wall deposition (tFIB), cortical parenchyma (cPAR) and the xylem part of the stem (sXYL) [8,29]. The genes specifically expressed in fibers were determined by contrasting data at the stage of intrusive elongation versus cortical parenchyma and at the stage of tertiary cell wall deposition versus the xylem part of the stem. The dynamics of gene expression during fiber development were interrogated by contrasting data at the stages of intrusive elongation and tertiary cell wall deposition. A total of 206 QTNs detected with GAPIT3 and 97 variants detected with GEMMA were associated with at least one gene differentially expressed in these comparisons (Tables S10 and S11). Figure 3 presents the number of differently expressed genes in QTN regions. Genes with $\left|log_{2}FC\right|\geq 2,\;padj0.01\leq 0.01$ were selected for further annotation based on the functions of orthologous genes in *Arabidopsis* and other plant species. Proceeding from annotation, the candidate genes fell into five groups: (1) genes related to the synthesis and modification of cell wall components; (2) genes controlling cell fate determination, growth and elongation; (3) genes associated with vesicular transport, intracellular trafficking of the cell wall components and solute transport across membranes; (4) genes involved in hormonal regulation; (5) genes associated with resistance to plant diseases (Table 1 and Table 2).

### 2.4. Candidate Genes Related to the Synthesis and Modification of Cell Wall Components

The flax primary cell wall is composed of cellulose microfibrils embedded in a hydrated matrix of hemicelluloses and pectin [107]. Hemicelluloses include xylans, mannans, mixed linkage β-glucans and xyloglucans. During fiber cell elongation and intrusive growth, the size and morphology of the cell changes driven by a coordinated series of biochemical reactions result in the biosynthesis and degradation of cell wall components [8]. Numerous enzymes participate in these processes to provide the remodeling of cell wall structure via selective synthesis and degradation of some polysaccharides. A number of candidate genes were predicted to be involved in the synthesis of cell wall carbohydrates. The *Lus10041644* gene within Chr4:15210519 QTN (Table 1) likely encodes fucosyltransferase 1, involved in xyloglucan synthesis [59]. In dicot plants, xylan consists of a linear backbone of β(1,4)-linked D-Xyl residues, some of which are substituted with α(1,2)-linked glucuronic acid (GlcA). The *Lus10033485* gene at Chr3:17134662 QTN was predicted to encode plant glycogenin-like starch initiation protein 1, which is glucuronyl transferase, responsible for the addition of GlcA residues onto xylan [48].

Several candidate genes were predicted to participate in the enzymatic modification of the cell wall polysaccharides. For example, *Lus10006462* and *Lus10030007* genes at QTNs Chr2:25126362 (Table 1) and Chr4:18493683 (Table 2), respectively, were predicted to encode endoglucanases capable of hydrolyzing glucans and callose [44,88]. Callose is a plant polysaccharide, which is localized to plasmodemata and also produced as a temporary cell wall constituent in response to stress [108]. Callose degradation may accompany the intercalation of elongating fibers, as they are simplastically isolated and have no plasmodemata [109]

Several genes, *Lus10030366, Lus10019631, Lus10013935* and *Lus10013936* at QTNs Chr4:1229010, Chr9:11359847 and Chr15:1632466 (Table 1), belonged to the alpha/beta-hydrolase protein superfamily, known to break carbon–carbon bounds [56]. The *Lus10043075* at Chr12:16047776 QTN was annotated to encode the O-glycosyl hydrolase family 17 protein with hydrolytic activity towards different types of hemicelluloses or callose [76]. *Lus10008974* at Chr9:1929944 was predicted to encode beta-galactosidase 12 [65]. In the flax tertiary cell wall, long galactan chains of the pectin rhamnogalacturonan-I (RG-I) are located between the cellulose microfibrils, thus preventing their lateral interaction. Beta-galactosidase shortens RG-I, which facilitates the lateral interaction of cellulose microfibrils and leads to maturation of the cell wall [7].

The flax primary cell wall also contains a variety of glycoproteins [110]. The QTN Chr13:1286479 (Table 1) harbored the *Lus10010666* gene, predicted to encode annexin, a soluble protein capable of Ca2+-dependent or -independent association with membrane phospholipids [79]. The *Lus10000957* gene (Table 2) within the Chr9:7152630 QTN encodes expansin-like B1, a cell wall loosening protein [79]. Class III peroxidases are cell wall localized proteins that can oxidize cell wall aromatic compounds within proteins and phenolics that are either free or linked to polysaccharides. Thus, they are also tightly associated to cell wall loosening and stiffening [40,41]. *Lus10013631*, *Lus10007051* and *Lus10029065* at QTNs Chr2:4683418 (Table 1), Chr3:17878746 (Table 1) and Chr4:9042565 (Table 2) were predicted to encode class III peroxidase. The Chr8:19022659 (Table 2) QTN was associated with the *Lus10002243* gene, which was annotated as encoding subtilase. Most subtilases are cell wall enzymes involved in the control of growth and development by regulating the properties of the cell wall and the activity of extracellular signaling molecules [91].

In flax fibers, the middle lamella is enriched in pectins, and rhamnogalacturonan-I is one of the constituents of the tertiary wall [8,107]. Pectin is synthesized in the Golgi apparatus, with most of the carboxyl groups methyl esterified, which results in pectin with low charge density. This likely increases the pectin flexibility and mobility in the wall. The transfer of methyl groups to pectin is catalyzed by methyltransferases [107]. *Lus10010138* at Chr1:27748943 QTN and *Lus10013945* at Chr15:1632466 QTN (Table 1) were annotated to encode methyltransferases, of which the former belonged to the S-adenosyl-L-methionine-dependent methyltransferase superfamily [34].

A a unique feature that provides strength to flax fibers is the presence of additional cell wall layers; of particular interest are candidate genes, the predicted function of which may be associated with secondary and tertiary wall deposition. The regulatory pathways implicated in secondary wall synthesis are well studied in *Arabidopsis* [111]. In this plant, the secondary cell wall develops in the xylary elements and fibers of the vascular system to provide mechanical strength to all plant organs. Among candidate genes, the *Lus10037568* gene at Chr3:25379963 QTN (Table 1) was annotated to encode class III homeodomain leucine zipper transcription factor (HD-ZIP III TFs) CORONA (CAN/AtHB150), which regulates the biosynthesis of secondary cell walls in *Arabidopsis* [49,112]. The *Lus10007528* and *Lus10007529* genes within QTN Chr9:971861 (Table 2) are orthologs to the *ATTBL33* gene encoding the TRICHOME BIREFRINGENCE-LIKE 33 protein, contributing to the deposition of secondary wall cellulose in *Arabidopsis* and, therefore, may be implicated in secondary cell wall synthesis in flax [92]. *Lus10024485* at Chr9:7487990 QTN (Table 2) was annotated as an ortholog of *A. thaliana* genes encoding the KNOTTED-like homeobox protein, which is a transcription factor involved in the secondary cell wall synthesis [96]. *Lus10025431* at Chr7:14140267 QTN (Table 1) probably encodes a small GTPase from the RAC/ROP family that is involved in the development of secondary cell walls of xylem vessels in *Arabidopsis* [61].

The flax secondary cell wall is composed of 30% lignin, a polymer made by cross-linking phenolic precursors, while the tertiary cell wall lacks this compound. A number of candidate genes may be involved in the synthesis and degradation of lignin. In particular, *Lus10041651* at QTN Chr4:1521051 and *Lus10026123* at Chr13:3759775 were predicted to encode enzymes of monolignol biosynthesis [80]. The *Lus10004043* gene at Chr4:17106278 (Table 2) encodes myb domain protein 20 that activates the lignin and phenylalanin biosynthesis genes during secondary wall formation [87]. The *Lus10007532* gene within QTN Chr9:971861 (Table 2) encodes laccase 5, which degrades lignin [93].

*Lus10029639* at Chr9:18121668 (Table 2) was predicted to encode the GDSL-like lipase/acylhydrolase superfamily protein. GDSL-like lipase/acylhydrolases are enzymes with broad substrate specificity and can hydrolyze many kinds of substrates such as thioesters, aryl esters, phospholipids and amino acids. In cotton, the GDSL lipase/hydrolase gene CotAD_74480 is expressed during secondary cell wall biosynthesis [98,99,100].

The orientation of the cellulose fibrils defines mechanical properties of flax secondary and tertiary cell walls. The direction of microfibrils in the wall is determined by movements of the cellulose synthase complex along the plasma membrane, partly guided by elements of the underlying cytoskeleton [107]. The *Lus10019171* gene at Chr3:4704531 QTN (Table 1) annotated as encoding microtubule-associated protein 70-2 may be involved in the control of the cellulose synthase movement [45].

### 2.5. Candidate Genes Controlling Cell Fate Determination, Growth and Elongation

In the plant embryo, the establishment of the apical–basal axis is followed by formation of the radial axis and finally of bilateral symmetry. The SAM arose between the two out-growing cotyledons [113]. *Lus10004377* at Chr9:5255847 QTN was predicted to encode the HD-Zip II transcription factor [66], which in *Arabidopsis* controls the embryonic apical patterning and the SAM function partly by interacting with HD-Zip III proteins, including ATHB15/CORONA implicated also in the control of the secondary cell wall biosynthesis [49,112]. The HD-Zip III genes are also involved in patterning along the radial and adaxial–abaxial axes of the embryo [113].

In *Arabidopsis* and other seed plants, the lateral roots are mainly derived from the pericycle cells located at the outermost cell layer of the stele along the roots. Plants can form lateral roots flexibly depending on environments due to the ability of the pericycle cells to maintain their cell division potential for a long time. *Lus10010638* at Chr13:12832925 (Table 2) was annotated as encoding a basic helix-loop-helix (bHLH) transcription factor. Its ortholog in *Arabidopsis PFB5* governs the competence of the pericycle cells to initiate the formation of the lateral root primordium [103].

Candidate genes *Lus10030216, Lus1002912, Lus10042531* and *Lus10010190* are probably involved in cell elongation. The *Lus10030216* gene associated with the Chr4:460524 QTN (Table 2) is an ortholog of the *ENOD3* gene, which in *Arabidopsis* encodes cytochrome P450 78A6 and controls seed size [52,53,54]. *Lus10002912* at the Chr3:26449997 QTN (Table 1) was predicted to encode the integrase-type DNA-binding superfamily protein, which is a SHINE transcription factor. SHINE transcription factors control the elongation of floral organs’ epidermal cells by affecting pectin metabolism [50]. Among their targets are genes from the CYP86A cytochrome P450 protein family. The *Lus10042531* gene at Chr9:17511839 QTN was annotated as the NAC047 transcription factor (Table 2). In *Arabidopsis*, *NAC047* controls the longitudinal cell expansion at the lower (abaxial) side of the leaf petiole [69]. The *Lus10010190* gene within the Chr9:693002 QTN (Table 1) is an ortholog of the *A. thaliana AT5G23210* gene for serine carboxypeptidase-like 34. In *Nicotiana tabaccum*, the extracellular type III carboxypeptidase controls cell elongation [64]. *Lus10023931* at Chr8:4371945 OTN (Table 2) may be involved in the control of cell differentiation. It was annotated to encode an RWP-RK domain family protein, which in *Arabidopsis* regulates the differentiation of the female gametophyte cells [89].

### 2.6. Candidate Genes Associated with Vesicular Transport, Intracellular Trafficking of the Cell Wall Components and Solute Transport across Membranes

Fundamental to cell elongation is the supplying of carbohydrates, proteins and other molecules to the new plasma membrane and cell wall. *Lus10013589* at QTN Chr8:1099671 (Table 1) was predicted to encode a SNARE protein critical for the movement of membrane and cargo proteins within the cell [62,114]. *Lus10030255* associated with Chr4:647773 (Table 1) is an ortholog of *AT3G61760* for DYNAMIN-like 1b required for the de novo assembly and maintenance of the plasma membrane during cell plate formation and cell expansion [55]. The *Lus10005850* gene within the Chr3:12697091 QTN (Table 2) likely encodes aldolase, which modulates V-ATPase-dependent vesicular trafficking events and actin cytoskeleton remodeling [82]. The *Lus10024499* gene associated with Chr9:7487990 QTN (Table 2) was annotated as calmodilin-binding protein IQD22, involved in cellular transport of specific cargo along microtubules [94,95]. The *Lus10027867* gene at Chr12:16474392 QTN (Table 2) was an ortholog of *AT3G19460*, encoding reticulon involved in endoplasmic reticulum–Golgi trafficking, vesicle formation and membrane morphogenesis [102].

Numerous QTNs were associated with genes encoding various transporters (Table 1 and Table 2). For cell wall expansion, sugar, potassium and malate transporters are of particular importance [115]. The *Lus10029506* gene at Chr4: 2,763,834 QTN (Table 1) was annotated to encode the K^+^/H^+^ transporter [57]. *Lus10042571* within the Chr9:17172377 QTN likely encodes aluminum malate transporter [68], and the *Lus10042516* gene contained within Chr9:17511839 QTN (Table 2) was predicted to encode sugar transporter [98]. A high concentration of osmotically active solutes results in an influx of water mediated by aquaporins. *Lus10010153* at Chr9:693002 QTN (Table 1) was predicted to encode NOD26-like intrinsic protein 5;1, an aquaporin [63]. Vacuolar invertase has long been considered as a major player in cell expansion. *Lus10016317*, harbored by QTN Chr2:2513654 (Table 1), encodes a cell wall/vacuolar inhibitor of fructosidase 1, an invertase that cleaves sucrose [36,37].

### 2.7. Candidate Genes Involved in Hormonal Regulation

Plant hormones regulate cell wall expansion and cell growth. Among them, auxin plays a vital role in controlling plant growth and development via promotion of cell proliferation, cell expansion and elongation, as well as cell differentiation [38]. Several candidate genes were either involved in auxin biosynthesis and transport or relayed regulatory signals to their targets via the auxin pathway. *Lus10000494* at QTN Chr9:1985807 (Table 1) likely encodes indole-3-pyruvate monooxygenase YUCCA8, catalyzing the last step in auxin biosynthesis, the oxidation of indole-3-pyruvic acid (IPA) to indole-3-acetic acid (IAA) [73]. *Lus10020193* at QTN Chr2:4360628 (Table 1) encodes the auxin efflux carrier family protein [38,39], while *Lus10029464* encodes the nodulin MtN21/EamA-like transporter family protein involved, in addition to auxin, in amino acid transport [84,85,86]. *Lus10029458*, *Lus10021553* (Table 2) and *Lus10004377* (Table 1) genes were predicted to encode transcription factors inducible by auxin [83,101]. *Lus10029458* was annotated as encoding a Dof transcription factor OBP3, which plays important roles in plant growth and development. In *Arabidopsis*, the expression level of the OBP3 protein increased following treatment with auxin, and transgenic plants overexpressing OBP3 showed a severe growth defect with altered root development and yellowish leaves [83]. The *Lus10004377* gene associated with Chr9:5255847 (Table 1) was predicted to encode HD-ZIP II transcription factor ATHB2, an early auxin-inducible gene in both *Arabidopsis* and wheat [66,113].

Cytokinins are a class of plant hormones that promote cell division in plant roots and shoots. The *Lus10010665* gene at the Chr13:1286479 QTN (Table 1) was predicted to encode UDP-glycosyltransferase 85A1, involved in cell cycle regulation by influencing a homeostasis of trans-zeatin, a cytokinin [77,78].

### 2.8. Candidate Genes Associated with Resistance to Plant Diseases

Plant cell wall alteration directly influences growth and stress response pathways [116]. This is especially relevant during plant responses to external stimuli. It is not surprising, therefore, that we found several abiotic-stress- and pathogenesis-related genes associated with QTNs for fiber traits. For example, a cluster of six genes, *Lus10007808–Lus10007814* within Chr8:18364995 QTN (Table 2), the *Lus10007455* gene at Chr9:8240615 QTN and the *Lus10020525* gene at Chr14:3502060 QTN (Table 2) were predicted to encode disease resistance TIR-NBS-LRR proteins [90].

Protein regulation is an important system to accomplish adaptation to different abiotic and biotic stresses. In plants, the ubiquitin-mediated system regulates various proteins in response to developmental signals and different environmental stresses [35]. Numerous candidate genes, namely *Lus10005814, Lus10005815, Lus10005818, Lus10013618, Lus10034490* and *Lus10039564* (Table 1), were predicted to encode RING/U-box superfamily proteins.

### 2.9. Network Analysis

**Figure 4:**
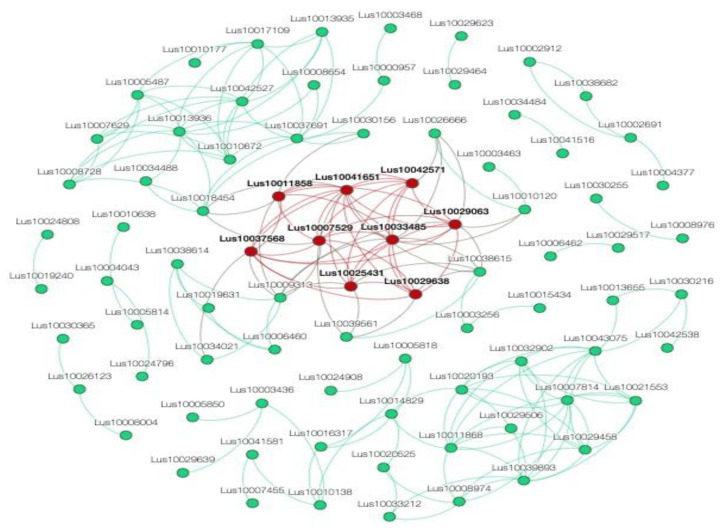
The network of most highly connected candidate genes. A node size is proportional to its adjacency value. Red node color defines gene membership in a subnetwork.

To provide further evidence for the involvement of the selected QTNs in trait variation, we constructed a co-expression network using candidate genes. Next, the network of most highly connected genes was obtained by filtering weighed network adjacencies with a threshold set to 0.9 (Figure 4). The annotation of the hub genes in this network highlighted the subnetwork of co-expressed genes, which contained genes involved in the secondary cell wall formation and modification (*Lus10033485, Lus10007529, Lus10025431, Lus10029638* and *Lus10037568)*, as well as two genes (*Lus10041651* and *Lus10011858*) (Table 1 and Table 2) which were predicted to encode lignin-synthesizing enzymes: cinnamoyl CoA reductase [60] and cytochrome B5 [75]. This subnetwork also included two genes for transporter proteins, *Lus10042571* and *Lus10029063*, which also may be involved in flax secondary cell wall formation. Interestingly, the Chr3:25379963 and Chr4:15210519 QTNs and their candidate genes *Lus10037568* and *Lus10041651* were about 85 kb and 160 kb away from the *uq.C3-1* and the *QPLH-Lu4.3* QTLs, respectively, both associated with plant height (Table S8b) [26,117]. Taking into account the low resolution of QTL mapping, we may speculate that genomic regions detected in GWAS and QTL analyses are in close proximity or even coincide.

## 3. Materials and Methods

### 3.1. Plant Material and Phenotyping

A total of 306 flax specimens containing 182 fiber flax accessions, 120 linseed flax accessions and 4 accessions of unknown morphotype were selected from the collection of the Federal Research Center for Bast Fiber Crops (Table S12). Linseed group was further subdivided into the following subgroups: 99 intermediate accessions, 5 large-seeded accessions and 16 crown accessions. The dataset included landraces, elite cultivars and breeding lines (Table S13). The geographic origin/release of the accessions encompasses 30 countries and all continents.

All plants were grown in the experimental field of Federal Research Center for Bast Fiber Crops in Torzhok, Tver region, Russia (57°02′ N, 34°58′ E; altitude: 165 m). A detailed description of plant growing and phenotyping was published in [10]. In brief, the accessions were planted using a completely randomized design in the spring of 2019 and two times in the spring of 2020 with a shift of two weeks. For each specimen, 20 seeds were sown in one hole. Ten rows of holes (200 sown seeds and 10 accession plots) alternated with a single row of standard flax varieties (controls), namely, Caesar and Severny cultivars, representing fiber and oil flax, correspondingly. Harvesting was carried out in the yellow ripening stage.

Phenotyping was performed in each planting. For replicate plots of each accession, we first measured 6 morphological traits informative of fiber content and quality: plant height, length of technical part of the stem, technical part weight, the diameter of the stem (at the top, at its middle part and at the butt), the number of internodes and distance between internodes. Next, the technical fiber was extracted manually from each stalk after retting. The isolated fiber was then weighed, and its content was assessed as a percentage of total straw weight, resulting in two additional phenotypic traits.

A challenging issue faced by flax growers and textile workers is the high labor intensity of methods for estimation of fiber content and predicting its quality. Consequently, three indirect indices of the prospective fiber quality assessment were introduced, which for each specimen enable to predict the proportion of fibrous matter in the stem and the feasibility of its isolation in the form of long fibers during mechanical processing. The stem slenderness index (SSI) is a ratio of the length of the technical part of the stem to its diameter. Plants with high SSI values are tall and contain high-quality fine fiber. The stem tapering index (STI) is the difference between the diameters in the lower and upper parts of the stem. The third index, the elementary fiber length (EFL), is calculated as the following:

(1) $$D_{f}=0.69D_{int}+0.096D_{tlength}+1.04D_{stem}-0.34$$

where $D_{int}$—average internode length of the stem (mm). The internode length is calculated as the ratio of the length of the technical part of the stem to the number of leaves:

$D_{tlength}$—technical length of the stem (mm);

$D_{stem}$—the diameter of the stem (the average between diameter values at the top, at its middle part and at the butt, mm).

We calculated all three indices in addition to morphology and fiber traits, bringing the total number of traits measured for each accession to eleven (Table S1).

### 3.2. DNA Sequencing and Variant Calling

The DNeasy Plant Mini Kit (Qiagen, Germantown, MD, USA) was used to extract DNA from collected leaves. The Illumina protocol generating paired-end reads of 150 bp was used to sequence DNA at the BGI (China). A total of 1143.850625 Gbytes of raw data comprising 7.626 billon reads with an average of 9.3× coverage or 3.7 Gbp per sample were generated. Reads were processed and aligned to NCBI flax reference genome assembly ASM22429v2 with bwa-mem using default parameters [118]. NGSEP [119] version 4.0. was used to call variants identified in the 3,416,829 biallelic SNPs. VCF tools [120] was applied to retain SNPs with minor allele frequency (MAF) > 5% and genotype call-rate > 85%. A total of 72,526 SNPs passed all filters and remained for further analysis.

### 3.3. Genetic Data Analysis

Principal component analysis (PCA) was performed using the PCA tools R package (Blighe internet). The linkage disequilibrium (LD) decay was evaluated using squared Pearson’s correlation coefficient (r^2^). The PopLDdecay [121] version 3.4.1 was run to calculate r^2^ in a 500 kb window. The LD decay was calculated based on r^2^ and the distance for each pair of SNPs using an R script in accordance with Hill–Weir approximation [122]. We applied Mann–Whitney–Wilcoxon test [123] to make group comparisons. Flax genome annotation [117] was kindly provided by Cloutier group (Ottawa Research and Development Centre).

### 3.4. GWAS

The univariate GWAS analysis was performed with GAPIT3 R package [32]. We selected the following models according to the workflow proposed by the package authors for multiple model testing: GLM, MLM, CMLM, FarmCPU, SUPER and Blink. The phenotype values were quantile normalized to a standard normal distribution. The FDR adjusted *p*-value threshold for candidate QTNs’ selection was set at 0.05.

Because of the high correlation of fiber traits, in addition to univariate GWAS models, a multivariate linear mixed model implemented in the GEMMA package [33] was applied. GEMMA tests marker associations with multiple phenotypes and controls both for population structure and for estimation of genetic correlation among complex phenotypes. A preliminary correlation analysis of 11 phenotypic traits using GEMMA selected five of them for further analysis: number of internodes (NI), technical length (TL), fiber weight (FW), plant height (PH) and fiber content (FC). The total number of single-nucleotide variants after filtering (call_rate 0.15, MAF 0.05) was 24,281 for 5 phenotypic traits. The threshold for determining statistically significant variants was 10^−6^.

### 3.5. Candidate Genes

A function of the candidate genes containing the identified QTNs was inferred from the function of their *Arabidopsis* orthologs in the TAIR database, from flax genome annotations kindly provided by Cloutier group (Ottawa Research and Development Centre, Canada) [117], as well as from the function of the homologous genes in other plant species, as described in the literature. We also searched for candidate genes within a window surrounding detected QTNs, where width was defined as proceeding from the LD decay estimated for each chromosome (Table S8).

### 3.6. Differential Gene Expression in Fiber Cells

The transcriptome libraries for bast fibers isolated at the stage of intrusive elongation (iFIB) and tertiary cell wall deposition (tFIB), cortical parenchyma (cPAR) and xylem part of the stem (sXYL) were obtained from SRA (BioProject PRJNA475325) as raw data in FASTQ format. The alignment to the flax reference genome version 2.0 was performed with STAR aligner [124] version 2.6.0c. The differential expression of genes was estimated with the DESeq2 [125] BioConductor package and the adjusted *p*-value threshold of 0.01.

### 3.7. Co-Expression Network Construction

To gain further insight into possible biological functions of the candidate genes, we used the transcriptome libraries described above (Section 3.6) to construct a co-expression network with the BioNERO package [126]. The package implements the Weighted Gene Co-expression Network Analysis (WGCNA) algorithm [127] to infer weighted networks from expression data. To estimate gene distances in the network, correlation values were raised to a power β = 6 that provided best fit to scale-free topology. The threshold 0.9 was used to filter weak weighed network adjacencies and highlight hub genes together with their connections. The network visualization was conducted in Gephi [128].

## 4. Conclusions

Genome-wide association studies use whole-genome sequencing information and are able to achieve higher precision in QTL detection in comparison with more conventional methods of QTL mapping. At the same time, dissecting GWAS loci to decipher the underlying biology is a labor-intensive multi-step process. High linkage disequilibrium between many variants often necessitates utilizing statistical fine-mapping approaches, which still have insufficient power to detect associations with alleles of small effect and low frequency and require the application of the FDR-controlling procedures for false discoveries and the use of functional genomic annotations for prioritization of variants before experimental testing. Recently, transcriptomics studies emerged as an approach to prioritize genomic regions for experimental validation of variants. In this study we integrated the results of GWAS and transcriptomics analyses to pinpoint genomic regions controlling fiber-related traits in flax. This approach revealed a number of genomic variants associated with different fiber traits, implying their complex and polygenic control. The variants mark potential candidate genes known to have a role in the synthesis and modification of cell wall components, in the control of cell fate determination, growth and elongation, in vesicular and intracellular trafficking of the cell wall components and solute transport across membranes, and in hormonal regulation and stress responses. Our results suggest tight genetic control of the fiber cell fate determination, as well as a leading role of auxin and cytokinins in promotion of fiber cell wall expansion and growth. The comprehensive set of QTNs identified in this study adds to better dissection of complex fiber traits, while the pinpointed candidate genes will be further used for in-depth investigations of their functional role and in marker-assisted selection.

## Figures and Tables

**Table 1:** Candidate genes predicted from the intersection of GAPIT3 R hits with data on gene expression in fibers.

QTN	Gene	Functional Annotation	*Arabidopsis* Gene Name	Reference	Predicted Function
Chr1:27748943	*Lus10010138*	S-adenosyl-L-methionine-dependent methyltransferases superfamily protein	*AT4G01240*	[34]	methylation and demethylation of cell wall proteins
Chr2:261056	*Lus10005814*	RING/U-box superfamily protein	*AT1G49230*	[35]	signaling proteins for receptor kinases, regulation of hormone signaling and abiotic/biotic stress response
	*Lus10005815*	RING/U-box superfamily protein	*AT1G49230*		
	*Lus10005818*	RING/U-box superfamily protein	*AT1G49230*		
Chr2:2513654	*Lus10016317*	cell wall/vacuolar inhibitor of fructosidase 1	*AT1G47960, ATC/VIF1, C/VIF1*	[36,37]	regulation of ABA response and salt tolerance
Chr2:4360628	*Lus10020193*	auxin efflux carrier family protein	*AT5G16530, PIN5*	[38,39]	regulates intracellular auxin homeostasis and metabolism
Chr2:4683418	*Lus10013618*	RING/U-box superfamily protein	*AT5G17600*	[35]	signaling proteins for receptor kinases, regulation of hormone signaling and abiotic/biotic stress response
	*Lus10013631*	peroxidase superfamily protein, class III	*AT5G1782,* *AtperoxP57*	[40,41]	cell wall localized proteins tightly associated to its loosening and stiffening
Chr2:4872459	*Lus10013655*	HSP20-like chaperones superfamily protein	*AT1G54400*	[42]	enhances membrane stability and detoxifies ROS by regulating the antioxidant enzymes system
Chr2:6885020	*Lus10038682*	major facilitator superfamily protein	*AT2G39210*	[43]	facilitates movement of small solutes across cell membranes
Chr2:25126362	*Lus10006462*	cellulase (glycosyl hydrolase family 5) protein	*AT1G13130*	[44]	hydrolyses the glycosidic bond
Chr3:4704531	*Lus10019171*	microtubule-associated proteins 70-2	*AT1G24764,* *ATMAP70-2*	[45]	outlining tracks of cellulose production during SCW formation and delivery of cell wall components
Chr3:5077352	*Lus10007972*	UDP-glucosyl transferase 89B1	*AT1G73880, UGT89B1*	[46]	
Chr3:5883916	*Lus10040598*	high-affinity K+ transporter 1	*AT4G10310, ATHKT1*	[47]	reducing Na^+^ toxicity through K^+^ uptake
Chr3:17134662	*Lus10033485*	plant glycogenin-like starch initiation protein 1	*AT3G18660, GUX1, PGSIP1*	[48]	glucuronyl transferase responsible for the addition of GlcA residues onto xylan and for secondary wall deposition
Chr3:17878746	*Lus10007051*	peroxidase superfamily protein, class III	*AT2G4148, AtperoxP25*	[40,41]	cell wall localized proteins tightly associated to its loosening and stiffening
Chr3:25379963	*Lus10037568*	homeobox-leucine zipper family protein/lipid-binding START domain-containing protein	*AT1G52150, ATHB15, CNA, ICU4*	[49]	confers positional information which is required to establish the number and pattern of vascular bundles in the stem, and is involved in repression of secondary cell wall development in *Arabidopsis*
Chr3:26449997	*Lus10002912*	integrase-type DNA-binding superfamily protein	*AT5G25390, SHN2*	[50]	TF, regulates floral organs’ epidermal cell elongation by affecting pectin metabolism
Chr4:184242	*Lus10030156*	glucose-1-phosphate adenylyltransferase family protein	*AT2G39770,* *CYT1, EMB101, GMP1, SOZ1, VTC1*	[51]	provides GDP-mannose, which is used for cell wall carbohydrate biosynthesis, including lignin and protein glycosylation
Chr4:466800	*Lus10030216*	cytochrome P450, family 78, subfamily A, polypeptide 6	*CYP78A6, EOD3*	[52,53,54]	Cell expansion, enhancer of DA-1 3 and seed and fruit development
Chr4:647773	*Lus10030255*	DYNAMIN-like 1B	*AT3G61760, ADL1B*	[55]	membrane trafficking, essential for plant cytokinesis and polarized cell growth
Chr4:1229010	*Lus10030366*	alpha/beta-hydrolases superfamily protein	*AT3G62860*	[56]	breaking of carbon–carbon bonds
Chr4:2763834	*Lus10029506*	cation/hydrogen exchanger 28	*AT3G52080, chx28*	[57]	cation/hydrogen exchange
Chr4:7789243	*Lus10039564*	F-box/RNI-like superfamily protein	*AT5G67140*	[58]	SCF ubiquitin ligase complex
Chr4:14879575 Chr4:14879658	*Lus10041581*	lysine histidine transporter 1	*AT5G40780, LHT1*	[30]	solute transporter
Chr4:15210519	*Lus10041644*	fucosyltransferase 1	*AT2G03220, ATFT1, ATFUT1, FT1, MUR2*	[59]	xyloglucan synthesis
	*Lus10041651*	cinnamoyl CoA reductase 1	*AT1G15950, ATCCR1, IRX4*	[60]	lignin biosynthesis
Chr7:14140267	*Lus10025431*	RAC-like 2	*AT5G45970,* *ATRAC2, ATROP7*	[61]	secondary cell wall of xylem vessels
Chr8:1099671	*Lus10013589*	syntaxin of plants 121	*AT3G11820,* *ATSYP121,* *ATSYR1, PEN1*	[62]	secretory traffic to the plasma membrane
Chr9:693002 Chr9:695025	*Lus10010153*	NOD26-like intrinsic protein 5;1	*AT4G1038, NIP5;1, NLM6, NLM8*	[63]	aquaporin
	*Lus10010190*	serine carboxypeptidase-like 34	*AT5G23210, SCPL34*	[64]	present in cell wall of elongating cells
Chr9:1929944	*Lus10008974*	beta-galactosidase 12	*AT4G26140, BGAL12*	[65]	plant cell wall remodeling
Chr9:5255847	*Lus10004364*	alpha/beta-hydrolases superfamily protein	*AT4G16820,* *PLA-I{beta]2*	[56]	breaking of carbon–carbon bonds
	*Lus10004377*	homeobox protein 2	*AT4G1678, ATHB2, HAT4, HB-2*	[66]	cell expansion and cell proliferation in the response to auxin
Chr9:8233979	*Lus10007455*	disease resistance protein (TIR-NBS-LRR class) family	*AT3G4448, cog1, RPP1*	[67]	plant immunity
Chr9:11359847	*Lus10019631*	alpha/beta-hydrolases superfamily protein	*AT1G72620*	[56]	breaking of carbon–carbon bonds
Chr9:17239226	*Lus10042571*	aluminum-activated malate transporter family protein	*AT5G46600*	[68]	transporter
Chr9:17408991	*Lus10042531*	NAC domain-containing protein 47	*AT3G0407, NAC047, SHYG*	[69]	NAC TF, localized longitudinal cell expansion
	*Lus10042537*	malectin/receptor-like protein kinase family protein	*AT3G04690, ANX1*	[70]	perception and relay processes at cell membranes
Chr9:18936691	*Lus10024808*	WUSCHEL-related homeobox 4	*AT1G46480, WOX4*	[71]	regulates the dynamic balance of division and differentiation of plant stem cells
	*Lus10024818*	cytochrome P450, family 81, subfamily D, polypeptide 5	*AT4G3732, CYP81D5*	[72]	defense response
Chr9:19858075	*Lus10000494*	flavin-binding monooxygenase family protein	*AT4G2872, YUC8*	[73]	indole-3-pyruvate monooxygenase YUCCA8, responsible for auxin biosynthesis in leaves
Chr9:20032070- Chr9:20032149	*Lus10011868*	CLAVATA3/ESR-RELATED 41	*AT3G24770, CLE41*	[74]	regulates vascular cell division, vascular organization and xylem differentiation in vascular tissue
	*Lus10011858*	cytochrome B5 isoform B	*AT2G32720, CB5-B*	[75]	lignin biosynthesis
Chr12:16047776	*Lus10043075*	O-glycosyl hydrolase family 17 protein	*AT5G20870*	[76]	hydrolytic activity towards different types of hemicelluloses or callose
Chr13:1286479	*Lus10010665*	UDP-glycosyltransferase superfamily protein	*AT1G2240, ATUGT85A1, UGT85A1*	[77,78]	cell cycle regulation, influences trans-zeatin (cytokinin) homeostasis likely through O-glycosylation
	*Lus10010666*	annexin 8	*AT5G12380,* *ANNAT8*	[79]	cell wall protein
Chr13:3759775	*Lus10026123*	hydroxycinnamoyl-CoA shikimate/quinate hydroxycinnamoyl transferase	*AT5G48930, HCT*	[80]	lignin biosynthesis
Chr15:1632466	*Lus10013935*	alpha/beta-hydrolases superfamily protein	*AT4G18550*	[56]	breaking of carbon–carbon bonds
	*Lus10013936*	alpha/beta-hydrolases superfamily protein			
	*Lus10013945*	O-methyltransferase family protein	*AT4G35160*		transfer of methyl groups to various biomolecules

**Table 2:** Candidate genes predicted from the intersection of GEMMA hits with data on gene expression in fibers.

QTN	Gene	Functional Annotation	*Arabidopsis* Gene Name	Reference	Predicted Function
Chr2:734912 Chr2:748984 Chr2:752999	*Lus10019240*	glycosyl hydrolase family protein	*AT5G10560*	[76]	hydrolysis of glycosidic bonds
	*Lus10019273*	basic leucine-zipper 6	*AT2G22850, AtbZIP6, bZIP6*		transcription factor
Chr2:3633762 Chr2:3633790	*Lus10003436*	glutamate receptor 2.7	*AT2G29120, GLR2.7*	[81]	non-selective cation channel
Chr2:6831421	*Lus10038682*	major facilitator superfamily protein	*AT2G39210*	[43]	facilitates movement of small solutes across cell membranes
Chr2:17384359 Chr2:17384362	*Lus10033212*	RAD-like 6	*AT1G75250, RL6, RSM3*		MYB family TF
Chr3:12697091	*Lus10005850*	aldolase superfamily protein	*AT4G26530*	[82]	modulates V-ATPase-dependent vesicular trafficking events and actin cytoskeleton remodeling
Chr4:460524	*Lus10030216*	cytochrome P450, family 78, subfamily A, polypeptide 6	*AT2G46660, CYP78A6, EOD3*	[52,53,54]	cell expansion, enhancer of DA-1 3, seed and fruit development
Chr4:2376445	*Lus10029458*	OBF-binding protein 3	*AT3G55370, OBP3*	[83]	TF, pays a role in plant growth and development, induced by SA and auxin
	*Lus10029464*	nodulin MtN21/EamA-like transporter family protein	*AT2G39510*	[84,85,86]	amino acid and auxin transporters
Chr4:9042565	*Lus10029065*	peroxidase superfamily protein, class III	*AT5G05340, AtperoxP52*	[40,41]	cell wall localized proteins tightly associated to its loosening and stiffening
	*Lus10029063*	major facilitator superfamily protein	*AT2G40460*	[43]	facilitates movement of small solutes across cell membranes
Chr4:17106278	*Lus10004043*	myb domain protein 20	*AT1G66230*	[87]	activates lignin and phenylalanine biosynthesis genes during secondary wall formation
Chr4:18493683	*Lus10030007* *Lus10030008*	glycosyl hydrolase 9B8	*AT2G32990, GH9B8*	[88]	membrane endo (1→4)-β-D-glucanase (cellulase), wall assembly and cell elongation, fruit ripening and floral abscission
Chr8:4371945 Chr8:4833196	Lus10034491	major facilitator superfamily protein	*AT4G34950*	[43]	facilitates movement of small solutes across cell membranes
	*Lus10023931*	plant regulator RWP-RK family protein	*AT2G17150*	[89]	control of cell differentiation
Chr8:18364995	*Lus10007808,* *Lus10007809,* *Lus10007810,* *Lus10007811,* *Lus10007813, Lus10007814*	disease resistance protein (TIR-NBS-LRR class) family	*AT5G36930*	[90]	plant immunity
Chr8:19022659	*Lus10002243*	subtilase family protein	*AT1G04110, SDD1*	[91]	cell wall enzyme
Chr9:971861	*Lus10007528,* *Lus10007529*	TRICHOME BIREFRINGENCE-LIKE 33	*AT2G40320, TBL33*	[92]	contribute to the synthesis and deposition of secondary wall cellulose, presumably by influencing the esterification state of pectic polymers
	*Lus10007532*	laccase 5	*AT2G40370, LAC5*	[93]	lignin degradation and detoxification of lignin-derived product
Chr9:7152630	*Lus10000957*	expansin-like B1	*AT4G17030, ATEXLB1, ATEXPR1, ATHEXPBETA 3.1, EXLB1, EXPR*	[79]	cell wall loosening
Chr9:7487990 Chr9:7488554	*Lus10024499*	IQ-domain 22	*AT4G23060, IQD22*	[94,95]	Ca^2+^/CaM-regulated scaffold for cellular transport of specific cargo along microtubules
	*Lus10024485*	KNOTTED-like homeobox of Arabidopsis thaliana 7	*AT1G62990,* *IXR11, KNAT7*	[96]	Secondary-wall-associated transcription factor
Chr9:8240615 Chr9:8240618	*Lus10007455*	disease resistance protein (TIR-NBS-LRR class) family	*AT3G44480, cog1, RPP1*	[90]	plant immunity
Chr9:9241973	*Lus10006152*	cytochrome P450, family 90, subfamily D, polypeptide 1	*AT3G13730, CYP90D1*	[72]	modification of cyclic terpenes and sterols in the brassinosteroid, abscisic acid and gibberellin pathways
Chr9:17172377 Chr9:17190919	*Lus10042571*	aluminum-activated malate transporter family protein	*AT5G46600*	[68]	transporter
Chr9:17511839 Chr9:17513033	*Lus10042531*	NAC domain-containing protein 47	*AT3G04070, anac047, NAC047*	[69]	transcription factor
	*Lus10042516*	sugar transporter 1	*AT1G11260, STP1*	[97]	H+/monosaccharide cotransporter
Chr9:18121668	*Lus10029638* *Lus10029639*	GDSL-like lipase/acylhydrolase superfamily protein	*AT1G54790*	[98,99,100]	growth, biotic stress response, in cotton the GDSL (GhGDSL) li pase/hydrolase gene (CotAD_74480) is expressed during SCW biosynthesis
Chr11:11843614	*Lus10021553*	auxin-responsive family protein	*AT3G25290*	[101]	TF, bind to AuxRE in the promoters of auxin-regulated genes
Chr12:16474392	*Lus10027867*	reticulon family protein	*AT3G19460*	[102]	endoplasmic reticulum-Golgi trafficking, vesicle formation and membrane morphogenesis
Chr13:12832925	*Lus10010638*	basic helix-loop-helix (bHLH) DNA-binding superfamily protein	*AT1G27660, PFA5*	[103]	governs the competence of pericycle cells to initiate lateral root primordium formation
Chr13:18496268	*Lus10030901*	cysteine-rich RLK (RECEPTOR-like protein kinase) 2	*AT1G70520, CRK2*	[104]	plant immunity
Chr14:3502060	*Lus10020525*	disease resistance protein (TIR-NBS-LRR class) family	*AT4G16950, RPP5*	[105]	plant immunity
Chr14:13328874	*Lus10032902*	HSP20-like chaperones superfamily protein	*AT1G54400*	[42]	enhances membrane stability and detoxifies ROS by regulating the antioxidant enzymes system
Chr15:124776	*Lus10007632* *Lus10007633*	cysteine-rich RLK (RECEPTOR-like protein kinase) 25	*AT4G05200, CRK25*	[106]	plant immunity

## Data Availability

The data that support the findings of this study are openly available at Figshare https://figshare.com/s/86a68ecfacf6872ef239 (accessed on 26 September 2022) (phenotypes) and at European Variation Archive (EVA) Project: PRJEB46073, Analyses: ERZ2775743 (genetic varants).

## Supplementary Materials

The following supporting information can be downloaded at: https://www.mdpi.com/article/10.3390/ijms232314536/s1.

## Appendix A

The following abbreviations were used for phenotyping traits:

Plant height	(PH)
Technical stem length	(TL)
Technical part weight	(TW)
Stem diameter	(SD)
Number of internodes	(NI)
Distance between internodes	(DI)
Fiber content	(FC)
Fiber weight	(FW)
Elementary fiber length	(ELF)
Stem slenderness index	(SSI)
Stem tapering index	(STI)
