# Supplementary figures and images for: Genetic Determinants of Fiber-Associated Traits in Flax Identified by Omics Data Integration
Source: Int J Mol Sci. 2022 Nov 22;23(23):14536. doi: 10.3390/ijms232314536 (PMC9738745; doi:10.3390/ijms232314536)

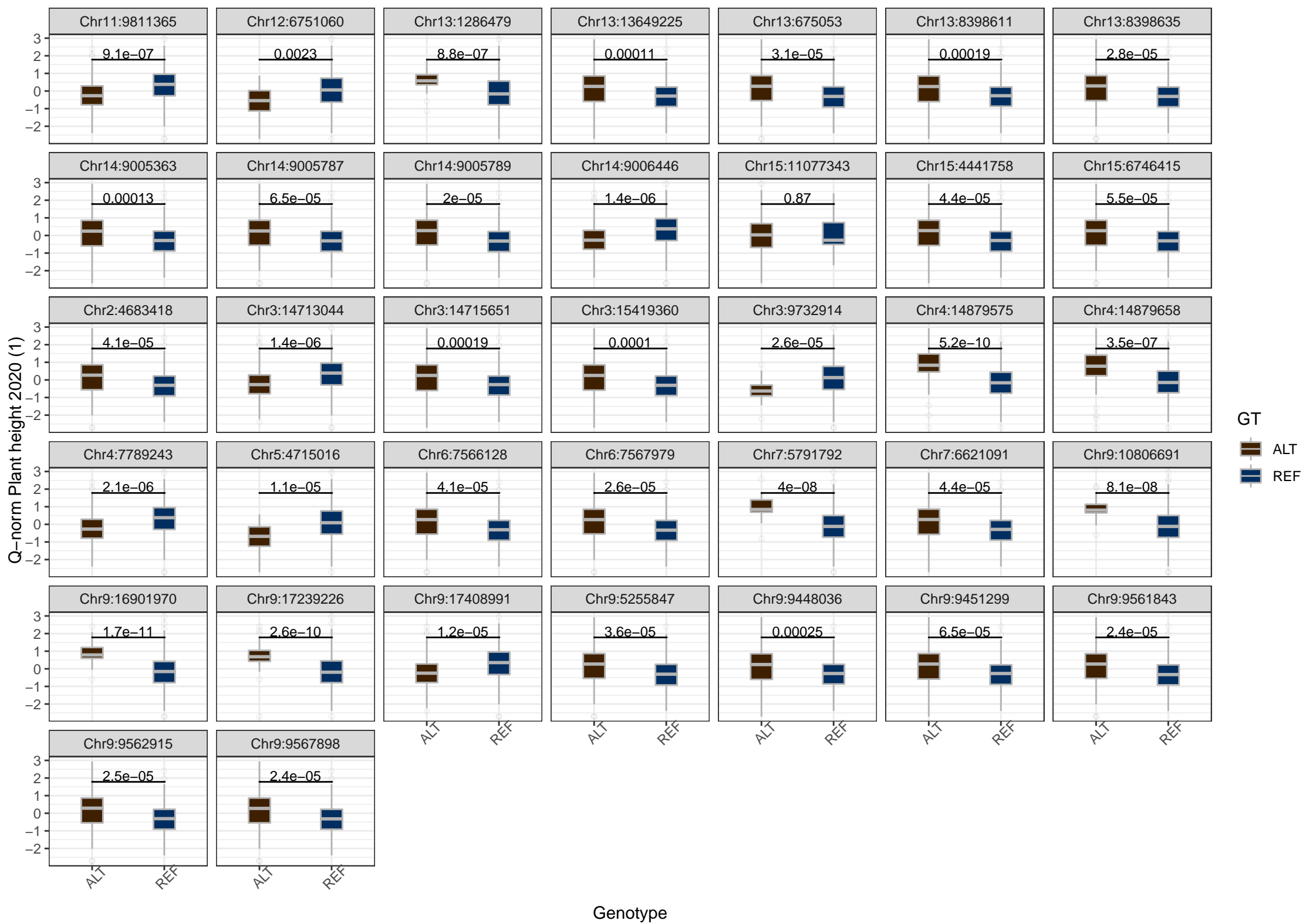

Supplement: Supplementary file 1 [file ijms-23-14536-s001.zip › Figure S1.pdf]

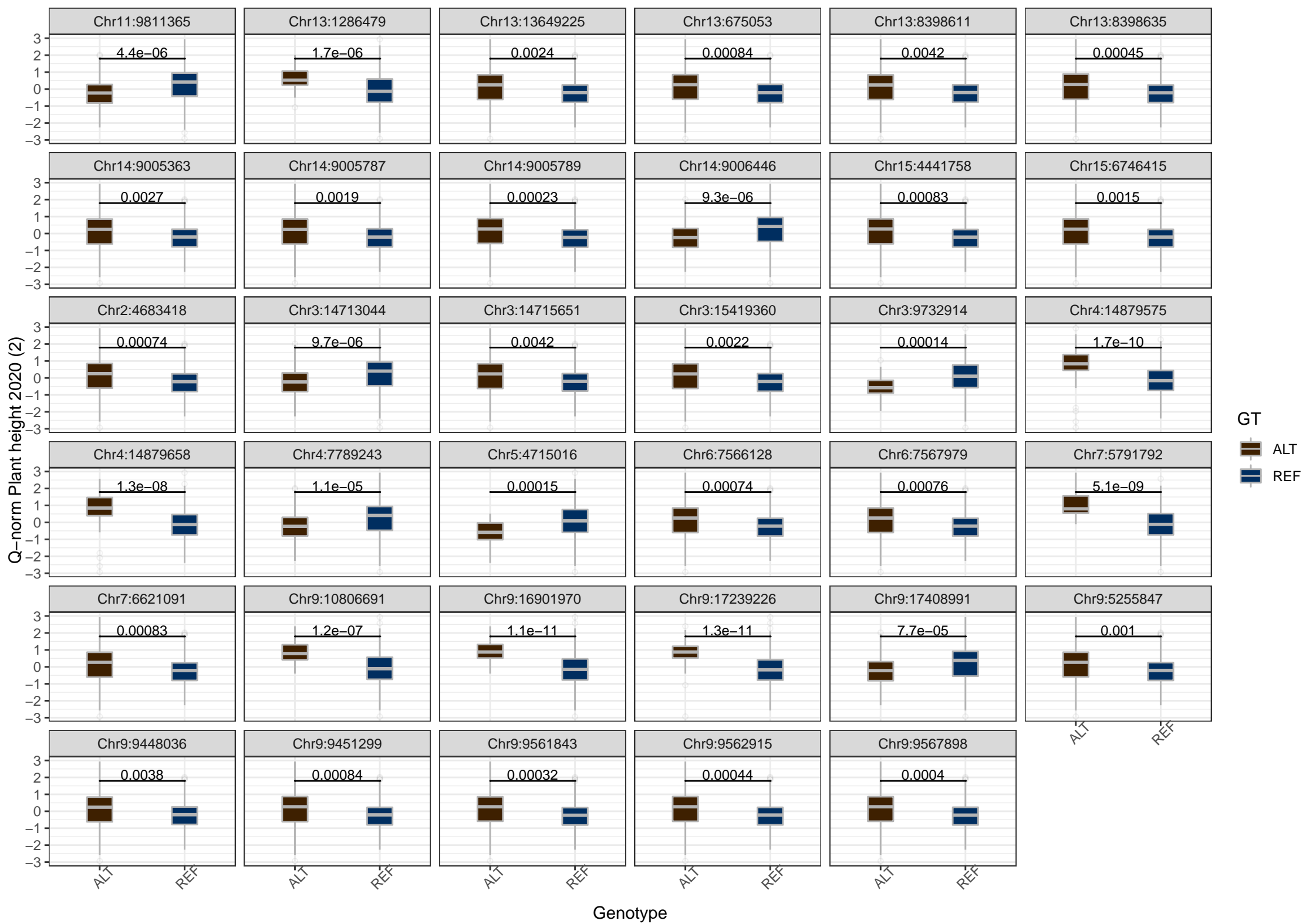

Supplement: Supplementary file 1 [file ijms-23-14536-s001.zip › Figure S2.pdf]
